# Acute Water Intoxication With Resultant Hypo-Osmolar Hyponatremia Complicated by Hypotension Secondary to Diffuse Third-Spacing

**DOI:** 10.7759/cureus.18410

**Published:** 2021-09-30

**Authors:** William Rizzuto, Norah Shemery, Josh Bukowski

**Affiliations:** 1 Emergency Medicine, San Antonio Military Medical Center, San Antonio, USA

**Keywords:** keywords: water intoxication, non-cardiogenic pulmonary edema, hypotonic hyponatremia, hypotension, symptomatic hyponatremia

## Abstract

Hypotonic hyponatremia secondary to acute water intoxication is most commonly associated with primary polydipsia in the setting of psychiatric illness. However, in certain circumstances, otherwise healthy individuals can be compelled to consume large enough volumes of water to overwhelm the kidney’s dilutional capacity of urine and cause a potentially life-threatening rapid decline in serum sodium. We present such a case of a 20-year-old basic military trainee with acute symptomatic hypotonic hyponatremia after drinking five to six liters of water prior to urine drug testing. The clinical manifestations of this disorder are non-specific and can be seen with many different pathologic processes, presenting a diagnostic challenge to the emergency clinician. This challenge can be further complicated by unclear or unobtainable history depending on clinical presentation. The authors will discuss key diagnostic and treatment elements of this potentially life-threatening disease to encourage clinicians to utilize social history when evaluating cases of acute water intoxication and resultant symptomatic hypotonic hyponatremia.

## Introduction

Hyponatremia is the most commonly identified electrolyte disturbance [1]. It is a clinical feature in 15-20% of emergency department admissions to hospitals and is associated with increased mortality, morbidity, and length of hospitalization [1]. It is defined as a decrease in the serum sodium concentration below 136 mmol per liter [2]. Hypotonic hyponatremia secondary to water intoxication is uncommon given the large amount of water required to consume to exceed the body's normal excretory capacity [2]. Symptoms of hypotonic hyponatremia are varied and dependent on whether it is acute or chronic in onset. The clinical manifestations are largely related to dysfunction of the central nervous system and are more severe when the decrease in sodium concentration is large, rapid, or both [2]. It is possible for mild to moderate symptoms of acute hyponatremia (i.e. lethargy, nausea, headache) to rapidly progress to severe symptoms of seizures and respiratory arrest [3]. Symptomatic patients must be monitored closely, and actions should be taken to gradually address their hyponatremia, especially if their hyponatremia is less than 125 mmol per liter [4-5]. Additionally, care must be taken to prevent the over-correction of symptomatic hyponatremia due to the risk of Osmotic Demyelination Syndrome (ODS) with rapid sodium correction [6]. We report a case of acute water intoxication that caused moderate hypo-osmolar hyponatremia and subsequent hypotension resulting from diffuse third-spacing of intravascular fluid.

## Case presentation

An otherwise healthy 20-year-old male basic military trainee presented to the emergency department (ED) via emergency medical services (EMS) with a one-day history of nausea, vomiting, and abdominal pain. The patient reported needing to provide a urine sample for a training program earlier in the day. Unable to provide a sample, he reportedly drank 5 to 6 liters of water until he could provide a sample. Within one hour of consumption, he became nauseated until he started vomiting and developed periumbilical/right lower quadrant abdominal pain. Additionally, he began to feel lightheaded and fatigued just prior to transportation to the ED.

EMS was called to transport the patient to the emergency department. EMS reported initial systolic blood pressure of 70 with a diastolic of 40 without compensatory tachycardia.

The patient was given two liters of intravenous lactated ringers by EMS without improvement in his blood pressure. Additionally, he was found to be hypoglycemic, with serum glucose of 58 mg/dL, and given one amp of dextrose-50 by EMS. On arrival to the ED, his vital signs were notable for systolic blood pressure or 82 with a diastolic of 54 and oxygen saturation of 89% on room air improved to 95% on two liters nasal cannula. The initial point of care glucose demonstrated persistent hypoglycemia with a glucose of 71 mg/dL. An additional amp of dextrose-50 was given. Physical examination was remarkable for pale appearing lethargic young adult male with moderate diffuse abdominal tenderness without rebound or guarding. Additionally, bibasilar crackles were appreciated on pulmonary auscultation. He was alert and oriented to person, place, and time. There were no focal neurologic deficits on examination. 

Venous blood gas was obtained upon patient arrival and was remarkable for sodium of 122 mmol/L, potassium of 3.5 mmol/L, and magnesium of 1.5 mmol/L. In the setting of persistent hypotension unresponsive to fluids, he was given 100 mg hydrocortisone without improvement in his blood pressure. He was given one liter of normal saline over one hour after identifying his low serum sodium as a possible contributing factor to his presenting symptoms. Hypertonic saline was not given in the setting of a normal neurologic examination and concern for alternative diagnoses at the time of the identification of his low serum sodium.

**Figure 1:**
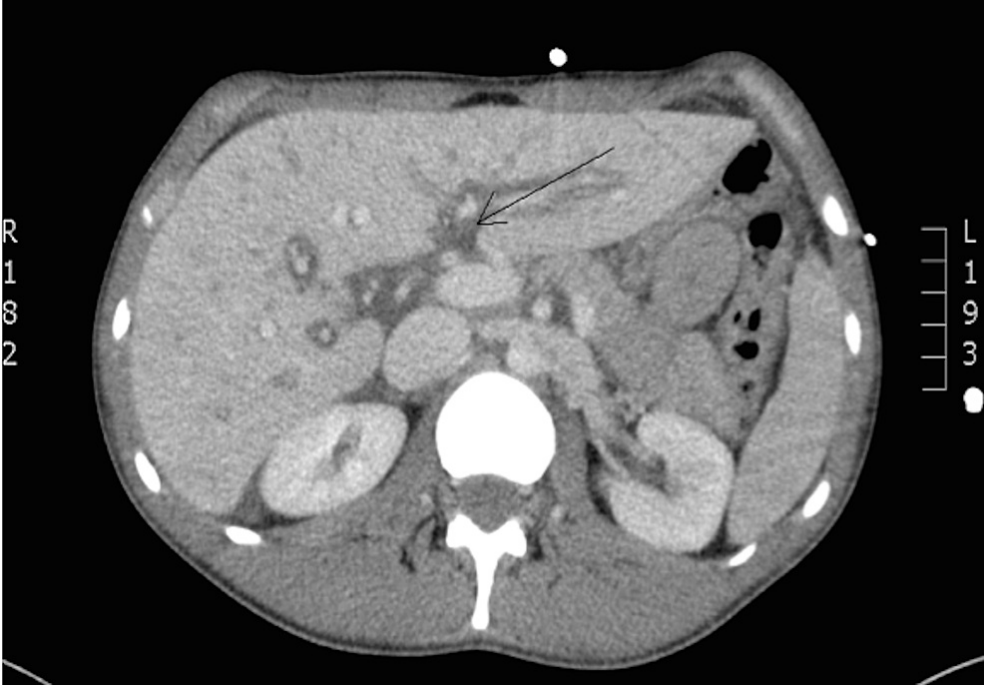
CT Abdomen and Pelvis with IV contrast demonstrating peri-portal edema. (black arrow)

**Figure 2:**
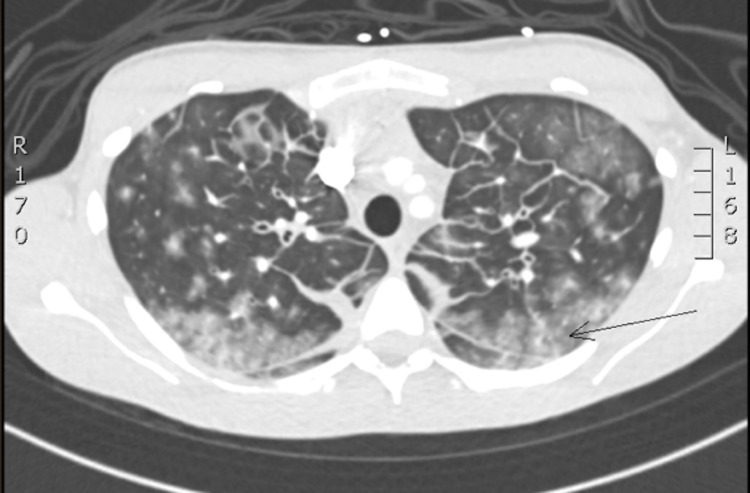
CT Chest Pulmonary Angiogram demonstrating non-cardiogenic pulmonary edema. (black arrow)

Computerized tomography (CT) scan of the abdomen and pelvis, and a CT chest pulmonary angiogram were obtained and notable for generalized intra-abdominal edema (Figure 1), and lung imaging consistent with non-cardiogenic pulmonary edema (Figure 2). This constellation of findings is consistent with non-specific third spacing pathology.

The patient was in the ED for a total of four hours. The initial differential diagnosis was broad and included: sepsis, pulmonary embolism, adrenal insufficiency, and symptomatic hyponatremia. After completion of the evaluation in the ED, it was determined that the etiology of his presenting symptoms was acute water intoxication complicated by moderate symptomatic hyponatremia with diffuse fluid third spacing and resultant hypotension and hypoxic respiratory failure. A repeat serum sodium of 124 mmol/L was obtained four hours after the initial venous blood gas. In consultation with the intensive care unit and internal medicine service, the patient was started on a five percent dextrose (D5) normal saline drip at 1.5 times maintenance and was admitted to an intermediate care unit. 

While inpatient, a urine osmolality of 111 mOsm/kg was obtained, which in conjunction with urine sodium less than 20 mmol/L is consistent with the patient's hypovolemia. Additionally, a serum osmolality of 264 mOsm/kg was obtained. These lab values are consistent with the diagnosis of hypo-osmolar hyponatremia complicated by diffuse third-spacing of intravascular fluid and resultant hypovolemia.

The patient's electrolytes and renal function were checked every four hours during his admission. Of note, his serum sodium rose to 133 mmol/L twenty hours into his admission, and D5-water was administered to prevent further over-correction of his serum sodium. Over a twenty-four-hour period, the total increase in his serum sodium was 11 mmol/L, slightly higher than the goal increase of 6-8 mmol/L. No complications of this over-correction were observed. He was admitted for a total of three days, and after the first twenty-four hours, his serum sodium gradually normalized. His symptoms of abdominal pain, nausea, vomiting, and lightheadedness resolved with the correction of his serum sodium. He was discharged on day three of hospitalization without complication.

## Discussion

There are many etiologies of hyponatremia, largely differentiated by serum osmolality and volume status [2]. Acute water intoxication causes hypotonic hypo-osmolar hyponatremia. Hypotonic hyponatremia secondary to excessive water intake, specifically in the setting of no known underlying psychiatric disorder, is an uncommon etiology of hyponatremia [7]. This is due to the large volume of water required to be consumed over a short period of time for an otherwise healthy individual to exceed the excretion capacity of the renal system. In normal individuals, a water load inhibits osmoreceptor stimulation of thirst and vasopressin release, allowing for dilution of the urine down to less than 50 mOsm/kg, resulting in rapid water excretion [8]. Based on an average solute load of 800 mOsm daily, most individuals can excrete up to 16 liters of water in a day [8]. Therefore, an otherwise healthy individual can drink 16 liters of water over a twenty-four-hour period before becoming hyponatremic [8]. Maximal dilution of urine prevents hyponatremia unless water intake is extraordinarily large, typically greater than 1 liter per hour [9]. The patient in the above case reportedly consumed 5 to 6 L of water in one hour, overwhelming his kidney's dilutional capacity and resulting in an acute dilutional hypotonic hyponatremia.

Although it is difficult to establish the timing and degree of a decrease in serum sodium, symptoms are more severe when the decrease in sodium concentration is large or rapid [2]. Currently, the generally accepted time-based definitions of acute versus chronic hyponatremia are less than forty-eight hours for acute and greater than forty-eight hours for chronic [10]. Sodium levels used to define mild, moderate, and profound hyponatremia are variable in published research. The threshold to define profound hyponatremia ranges from 110 to 125 mmol/L, however studies have shown symptoms to be more common among patients with sodium concentrations below 125 mmol/L, especially if the decline was rapid [4-5].

Moderately symptomatic hyponatremia is defined as any biochemical degree of hyponatremia in the presence of moderately severe symptoms including nausea, confusion, and headache. More severe symptoms include vomiting, cardiorespiratory distress, deep somnolence, seizures, and Glasgow Coma Scale less than or equal to 8 [1]. With most clinical manifestations of disease, there is not a clear distinction between moderate and severe symptoms. Rather, there is a continuous spectrum of disease severity. A previous case series looked at seven cases of hyponatremia in marathon runners and identified an association between non-cardiogenic pulmonary edema and increased intracranial pressure [3]. In the case described above we see non-cardiogenic pulmonary edema without clinical evidence of increased intracranial pressure or cerebral edema. This suggests a spectrum of diseases with variable progression of symptoms.

Once symptomatic hyponatremia is identified correction of serum sodium should be initiated. This can be accomplished with normal saline in the emergency department. Special care must be taken when correcting a patient’s serum sodium concentration due to the risk of ODS with rapid sodium correction [6]. Observational studies have shown an increased risk of ODS in rapid serum sodium correction inpatient with pre-correction sodium concentrations of less than or equal to 120mmol/L [10]. The rate of increase of serum sodium concentration with sodium-containing fluid administration is not well defined and subject to multiple variables. A prospective observational study of fifty-eight patients with euvolemic hyponatremia observed a mean increase in serum sodium of 2 mmol/L with administration of 100mL of 3% saline, a sodium content equivalent to roughly 330mL of Normal Saline (NS) [11]. It is unlikely that a dangerous degree of increase in serum sodium will be seen with administration 100 to 250mL of 3% saline or 500 to 1000mL of NS in the symptomatic patient. There have been suggestions in the literature of ways to safely increase serum sodium concentrations with goals of increasing serum sodium concentration of 6 to 8 mmol/L in 24 hours, 12 to 14 mmol/L in 48 hours, and 14 to 16 mmol/L in 72 hours [12].

## Conclusions

Due to its potentially life-threatening complications, it is important for emergency clinicians to use a focused social history to consider the diagnosis of acute symptomatic hypotonic hyponatremia. There are certain patient populations at higher risk of acute water intoxication and resultant hypotonic hyponatremia, such as individuals who recently underwent drug testing, or marathon runners may inadvertently put themselves at risk for developing life-threatening hypotonic hyponatremia. Clinicians should maintain a high index of suspicion for acute water intoxication in all patients with supporting history and symptoms of cerebral dysfunction to include lethargy and confusion. Rapid assessment and diagnosis are crucial in the management of acute moderate to severe hypotonic hyponatremia to prevent progression of the disease. It is possible to safely initiate correction of low serum sodium concentrations in the symptomatic Emergency Department patient and prevent the possible life-threatening and irreversible neurologic sequelae of overcorrection.
